# Rapid Detection of *Mycobacterium tuberculosis* in Pleural Fluid Using Resuscitation-Promoting Factor-Based Thin Layer Agar Culture Method

**DOI:** 10.3389/fmicb.2022.803521

**Published:** 2022-02-16

**Authors:** Fengjiao Du, Aiying Xing, Zihui Li, Liping Pan, Hongyan Jia, Boping Du, Qi Sun, Rongrong Wei, Zhongquan Liu, Zongde Zhang

**Affiliations:** Beijing Key Laboratory of Drug Resistance Tuberculosis Research, Beijing Chest Hospital, Beijing Tuberculosis and Thoracic Tumor Research Institute, Capital Medical University, Beijing, China

**Keywords:** *Mycobacterium tuberculosis*, resuscitation-promoting factor, thin layer agar, pleural effusion, culture

## Abstract

**Background:**

Pleural tuberculous is difficult to diagnose. Culture is still considered the gold standard, especially in resource-limited settings where quick, cheap, and easy techniques are needed. The aim of the study was to evaluate resuscitation-promoting factors (Rpfs)-based thin layer agar (TLA) culture method for quick detection of *Mycobacterium tuberculosis* in pleural fluid.

**Methods:**

Patients with suspected pleural TB were enrolled prospectively in our hospital, pleural fluid of all patients were collected, stained with Ziehl–Neelsen for acid-fast bacilli (AFB), cultured on Rpfs-TLA, TLA, and Löwenstein–Jensen (LJ) medium, and identified according to recommended procedures.

**Results:**

A total of 137 suspected pleural TB were enrolled and categorized, including 103 pleural TB (49 confirmed and 54 probable pleural TB) and 34 non-TBP patients. The sensitivity of Rpfs-TLA for total pleural TB was 43.7% (34.5∼53.3%), higher than that of TLA 29.1% (21.2∼38.5%) and LJ 26.2% (18.7∼35.5%) (*p <* 0.01), and all specificity was 100% in the diagnosis of pleural TB. Median time to detection of a positive culture was 11.8 days (95% CI 10.4∼13.4) for Rpfs-TLA, 21.0 days (95% CI 19.1∼22.9) for TLA, and 30.5 days (95% CI 28.5∼32.5) for LJ (*p* < 0.001).

**Conclusion:**

Rpfs-TLA is an accurate, rapid, cheap, and easy culture method, which makes it promising for use in clinical laboratories.

## Introduction

Tuberculosis (TB) is one of the infectious diseases with the highest morbidity and mortality in the world, with an estimated 9.87 million new cases and 1.50 million deaths in 2020. China is ranked second after India, worldwide, with 842,000 new cases of TB and 30,000 deaths among human immunodeficiency virus-negative TB every year (impact of the COVID-19 pandemic on TB) (World Health Organization [WHO]., 2021). Fifteen percent of the total 7.0 million reported TB cases were extrapulmonary, among which pleural TB was the second most common type after lymph node TB and the most common cause of pleural effusion, especially in high TB burden areas (Liam et al., 2000; Vorster et al., 2015; World Health Organization [WHO]., 2021).

The definite diagnosis of pleural TB depends on the detection of *Mycobacterium tuberculosis* (*M.tb*) from the pleural effusion, sputum, or pleural tissue (Gopi et al., 2007; Vorster et al., 2015). However, due to the paucibacillary nature of the disease and/or non-replicating dormant bacteria that exists, reducing growth potential of these organisms in pleural effusion (PE) (Mukamolova et al., 2010; Rosser et al., 2017; Shaw et al., 2018; Dusthackeer et al., 2019), traditional microbiological methods are poor and long time consuming, for example, microscopy of pleural fluid and Ziehl–Neelsen staining are rarely positive (0–5%), and culture has more sensitivity of 24–58%, but takes 4–8 weeks to return a result (Barbas et al., 1991; Gopi et al., 2007; Trajman et al., 2008). Although using liquid culture media or BACTEC system can improve the sensitivity of 30–70% and a reduced report time (about 2 weeks), expensive equipment and reagents limit its widespread adoption (Maartens and Bateman, 1991; Shaw et al., 2018). Pleural biopsy with a high positive rate of 69.6–82% is not widely used as it is invasive, operator dependent, and technically difficult (particularly in children) (Casalini et al., 2018). Besides, molecular tests like the Xpert MTB/RIF assay could report results within a few hours, but show poor sensitivities (22.7–51.4%), are expensive, and require special expertise (Sehgal et al., 2016; Kaso and Hailu, 2021). Therefore, a rapid, simple, reliable, and cheap diagnostic test is urgently needed for pleural tuberculosis.

The ability of the host immune system to stimulate the transition of *M.tb* to a non-replicating state that requires external stimulation to reinitiate growth is well recognized (Biketov et al., 2000; Mukamolova et al., 2010; O’Connor et al., 2015). Resuscitation-promoting factors (Rpfs), acting as bacterial cytokines, are initially characterized through their resuscitating effect on non-replicating cells *in vitro* and *in vivo* through lysozyme and peptidoglycan hydrolase effects (Biketov et al., 2000; Kana et al., 2008; Gupta and Srivastava, 2012; Dusthackeer et al., 2019). There are five functionally redundant Rpf-like proteins (RpfsA–E) found in *M.tb*, originally discovered to restore culturability from a dormant state and also stimulate growth of viable bacteria, acting at concentrations of picomole or less (Mukamolova et al., 1998; Kana et al., 2008). They were later provided as preliminary evidence of the effect of mycobacteria in clinical samples (Mukamolova et al., 2010; Rosser et al., 2017; Dusthackeer et al., 2019). Although the precise mechanism by which Rpfs effect their growth-stimulatory action remains unknown, we hypothesized that the positive rate could be increased, and the report time should be shortened if the dormant *M.tb* in pleural effusion were resuscitated by Rpfs. Furthermore, thin layer agar (TLA) has been described as a simple, rapid, and inexpensive method allowing initial identification of *M.tb* based on colony morphology visualized microscopically and by incorporation of para-nitrobenzoic acid (PNB) in the medium (Idigoras et al., 1995; Martin et al., 2009a; Ardizzoni et al., 2015). Most of the studies on TLA come from developed countries or from high HIV prevalence settings (Martin et al., 2009b; Satti et al., 2010; Ardizzoni et al., 2015). Therefore, we performed a prospective study to evaluate the performance of resuscitation-promoting factor-based TLA culture method for quick detection of *Mycobacterium tuberculosis* in pleural effusion in China.

## Materials and Methods

### Ethical Approval

The ethical approval for this study was obtained from the Beijing Chest Hospital Ethics Committee (ethical approval no. BJXK-2015-08). Written informed consent was acquired from each participant.

### Study Design and Participants

Patients with evidence of pleural effusion demonstrated by X-ray and suspected of having pleural tuberculous were enrolled from July 2016 to November 2018 at the Beijing Chest Hospital. A minimum of 100 ml of pleural effusion volume of all patients was collected, stained for acid-fast bacilli (AFB), cultured on Rpfs-TLA, TLA, and Löwenstein–Jensen (LJ) medium, and identified according to recommended procedures. Routine clinical, biochemical, molecular biological, and microbiological examinations in pleural biopsy specimens or pleural fluid, histopathological examinations in pleural biopsy specimens, and acid-fast bacterium (AFB) staining in pleural biopsy specimens or sputum were collected over a follow-up period of at least 3 months. Individuals were excluded if they have previous tuberculosis history and tuberculosis contact history, or they have received antituberculosis therapies more than 2 weeks before enrollment. Major clinical characteristics of recruited subjects are summarized in Table 1.

**TABLE 1:** Clinical characteristics in the study groups.

Characteristics	Total (*N* = 137)	Pleural TB (*N* = 103)	Non-TB (*N* = 34)	*p*-value
Age, years, mean (range)	49 (18–86)	46 (18–86)	59 (26–79)	**<0.001**
Male sex (%)	83 (60.58)	64 (62.1)	19 (55.9)	0.548
**Pleural effusion test**				
ADA, U/L	43.41 ± 3.01	60.06 ± 25.75	17.63 ± 10.45	**0.023**
Lymphocyte,/μL	77.85 ± 31.18	79.12 ± 30.87	70.24 ± 26.93	**0.036**
Rivalta test*, (n)	95 (69.34)	70/103 (67.96)	25 (73.53)	0.669
Albumin, mg/dl	46.39 (38.21–51.86)	46.92 (41.40–52.73)	45.10 (36.43–51.94)	0.173
LDH, IU/L	393 (254–713)	444.10 (259–832)	335 (241–575)	**0.008**
CRP, mg/ml	26.85 (7.43–17.02)	33.55 (11.92–46.43)	15.32 (6.70–21.66)	**0.027**
**Underlying disease**				
Diabetes mellitus (%)	11 (8.03)	9/103 (8.73)	2/34 (5.88)	0.595
**Effusion site**				
Right	75 (54.74)	55 (53.40)	20 (46.51)	0.448
Left	53 (38.69)	37 (35.92)	16 (37.21)	0.883
Bilateral	18 (13.14)	11 (10.68)	7 (16.28)	0.348

*TB, tuberculosis; ADA, adenosine deaminase; LDH, lactate dehydrogenase; CRP, C-reactive protein.*

*Rivalta test is used as a puncture fluid test for differentiation of exudate and transudate.

*Bold values represent statistically significant (P < 0.05) when compared with pleural TB.*

### Definitions and Diagnosis

Patients were divided into three groups according to the previously published definition. (1) Definite pleural TB: For clinical specimens (including pleural effusion, sputum, or pleural biopsy tissue) that yielded positive *M.tb* by etiology (including bacteriology and molecular biology), or caseating granulomas were present in biopsy or surgical specimens, Rpfs-TLA culture result was not included. (2) Probable pleural TB: did not fulfill the criteria for bacteriologic confirmation but had been diagnosed with active pleural TB by a physician according to clinical findings, thoracoscopic reports, radiologic imaging, and 3 months of follow-up outcome since the date of enrollment. (3) Patients were designated as the control group (non-pleural TB), if other diagnoses except tuberculosis were made by microbiologic, histopathological, or serologic examinations, and the patient improved without receiving antitubercular treatment.

### Specimen Collection and Processing

A total of 100 ml of pleural effusion was collected in sterile tubes from each patient by laboratory personnel blinded to the clinical data of the patients and then centrifuged at 3,000 × *g* for 15 min at 4°C. The supernatant was discarded, leaving a 10-ml precipitation, and 10 ml of 4% (weight/volume) sodium hydroxide (NaOH) was added with a pipette, gently blowing until well blended, then kept at room temperature for 15 min for decontamination, and it was ensured that these operations were performed for no more than 20 min before centrifugation for 20 min at 3,000 × *g*. After the centrifugation, the supernatant was discarded immediately, which, according to experience and standard operating procedures, ensures complete digestion without killing the bacteria (Forbes et al., 2008). Finally, the sediment was resuspended in 0.5 ml of phosphate buffer, and 0.1 ml of the processed sample was inoculated and submitted for every culture with Rpfs-TLA, TLA, Löwenstein–Jensen (LJ) medium, and acid-fast bacilli smear.

### Acid-Fast Bacilli Smear Microscopy

Smear microscopy was performed according to the Clinical and Laboratory Standards Institute (CLSI) M48-A guidelines (Forbes et al., 2008). Specimens were stained for acid-fast microscopic examination using the Ziehl–Neelsen stain (BaSO Diagnostics Inc., Zhuhai, China). Smear-positive specimens were judged (graded from 1+ to 4+) according to the American Thoracic Society scale.

### Mycobacterial Culture and Detection

Standard procedures were performed for mycobacterial culture and smear microscopy. After decontamination, the resuspended samples were subjected to cultivation on solid Lowenstein–Jensen medium (Encode Medical Engineering Co., Ltd.), 7H11 (Becton, Dickinson, and Company) agar plates directly, and 7H11 agar plates were inoculated with *M.tb* resuscitation factor recombinant protein at 2 nmol per milliliter of Rpf B(Rvl009) and 2 nmol per milliliter of Rpf E(Rv2450c) (Zhongquan et al., 2007). For each culture method, 0.1 ml of sterile saline was added as negative control and a 0.1 ml of *M.tb* standard strain H37Rv (ATCC 27294) as positive control for quality control, then incubated at 37°C for up to 8 weeks in an incubator (Thermo Scientific Forma Reach CO_2_ Incubator, Thermo Fisher Scientific, Waltham, MA, United States), and TLA and Rpfs-TLA cultures were incubated for up to 6 weeks to record culture status and days to positivity (DTP).

All culture positive samples were subjected to Ziehl–Neelsen staining to confirm the presence of acid-fast bacilli. Then the cultivate individual colony preparation and PCR amplification were sequenced by RuiboBioTech (Beijing, China). DNA sequences were aligned with the IS6110 homologous sequences of the reference strain *M. tuberculosis* H37Rv. The identification was carried out in accordance with the TB Laboratory Diagnostic Test Procedure, published by the National Defense Tuberculosis Association in 2006.

### Statistical Analyses

Baseline clinical characteristics and demographic data analysis was performed using SPSS, version 17.0 (SPSS, Inc., Chicago, IL, United States). Categorical data were compared by Pearson’s Chi-square or Fisher’s exact test. Continuous variables were compared using non-parametric Mann–Whitney *U*-test. The sensitivity, specificity, positive predictive value (PPV), negative predictive value (NPV), likelihood ratio positive (LR+), and likelihood ratio negative (LR−) of different assays were calculated against the reference standard. Ninety-five percent confidence intervals (95% CI) were estimated according to the binomial distribution. All *p*-values reported were calculated two tailed with statistical significance set to *p*-value < 0.05.

## Results

### Patient Characteristics

**FIGURE 1:**
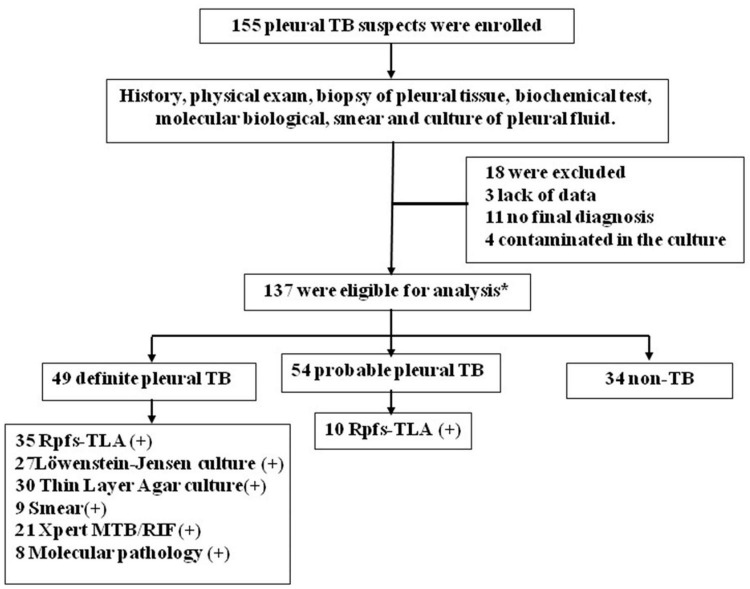
Flowchart of the study population. *Patients who were classified according to composite reference standard criteria that does not include. TB, tuberculosis; Xpert, Xpert MTB/RIF; Rpfs-TLA, resuscitation-promoting factor-based thin layer agar; Smear, acid-fast bacilli smear microscopy.

In this study, 155 participants with clinically suspected pulmonary TB were prospectively recruited. Eighteen patients were excluded from the study, 3 due to lack of data, 11 with no final diagnosis, and 4 contaminated in the culture. The remaining 137 participants with HIV-negative results were ultimately included for analyses (Figure 1). Based on the composite reference standard (in the methodology), including clinical, histopathologic, laboratory, and radiologic examinations and 3-month follow-up data, finally, 103 patients were designated as pleural TB (75.2%) (including 49 definite cases and 54 probable cases) and 34 patients without TB (24.8%) as controls. All patients with definite and probable pleural TB were considered to have pleural TB for calculation of sensitivity and specificity. The 34 patients without TB included 27 lung cancer (21 adenocarcinoma, 10 small cell, and 6 squamous cell carcinoma), 5 bacterial pleurisy, and 2 pleural mesotheliomas. The baseline demographic and clinical characteristics of the patients are summarized in Table 1. Pleural TB patients were significantly younger than non-TBP patients (*p*-value < 0.001). Adenosine deaminase (ADA) activity, lymphocyte count, lactate dehydrogenase (LDH), and C-reactive protein (CRP) of pleural effusion in pleural TB group were higher than in the no pleural TB group (all *p*-values < 0.05).

### Diagnostic Performance of the Resuscitation-Promoting Factors-Thin Layer Agar for Pleural Tuberculosis

**FIGURE 2:**
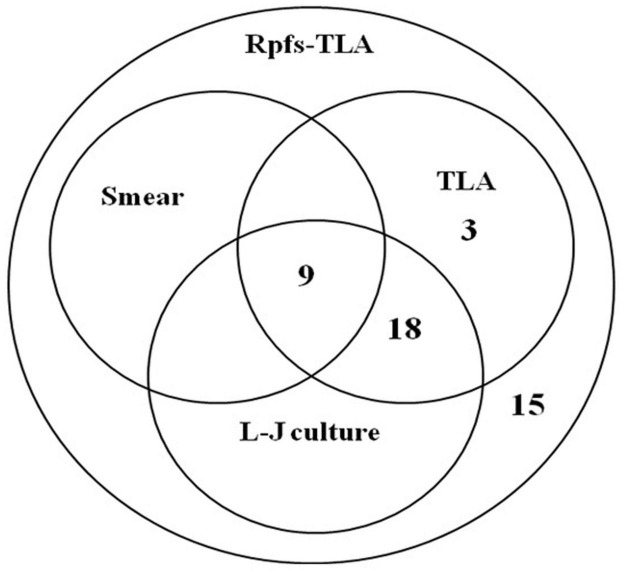
Venn diagram of the overlap among different diagnostics for pleural fluid testing. Rpfs, resuscitation-promoting factors; TLA, thin layer agar; L-J, Löwenstein–Jensen medium; Smear, acid-fast bacilli smear microscopy.

We first evaluated Rpfs-TLA testing of pleural fluid among the definite and probable pleural TB patients. The direct head-to-head performance comparison for *M.tb* detection showed that the sensitivity of Rpfs-TLA was 43.69% (45 of 103), higher than TLA at 29.13% (30 of 103, *p*-value < 0.05), Löwenstein–Jensen culture at 26.21% (27 of 103, *p*-value < 0.05), and smear at 8.74% (nine of 103, *p*-value < 0.001) (Figure 2). The sensitivity of TLA was slightly higher than the Löwenstein–Jensen culture, but not reaching statistical significance (*p*-value > 0.05). Smear had the lowest sensitivity. The specificity of all the four bacteriological methods was 100% (Table 2). Among 49 definite pleural TB cases, the sensitivities of Rpfs-TLA, TLA, and Löwenstein–Jensen culture were 71.43% [95% confidence interval (CI) = 57.59–82.15%], 61.22% (95% CI = 47.25–73.57%), and 55.10% (95% CI = 41.32–68.15%), with no significant difference among the three groups (all *p*-values > 0.05).

**TABLE 2:** Diagnostic performance of the smear, Löwenstein–Jensen, thin layer agar (TLA), and resuscitation-promoting factors (Rpfs)-TLA cultures in pleural TB.

Methods	Sensitivity %(95% CI)	Specificity %(95% CI)	PPV %(95% CI)	NPV %(95% CI)	LR + (95% CI)	LR- (95% CI)	Accuracy (95% CI)
Smear	8.74 (4.67–15.78)	100 (89.85–100)	100 (70.09–100)	26.56 (19.68–34.82)	-	91.3 (86–96.9)	31.39 (24.21–39.58)
Löwenstein–Jensen	26.21 (18.69–35.45)	100 (89.85–100)	100 (87.54–100)	30.91 (23.04–40.07)	-	73.8 (65.8–82.8)	44.53 (36.47–52.88)
TLA	29.13 (21.23–38.52)	100 (89.85–100)	100 (88.65–100)	31.78 (23.72–41.10)	-	70.9 (62.6–80.2)	46.72 (38.56–55.05)
Rpfs-TLA	43.69 (34.51–53.32)	100 (89.85–100)	100 (92.13–100)	36.96 (27.80–47.16)	-	56.3 (47.5–66.8)	57.66 (49.29–65.62)

*PPV, positive predictive value; NPV, negative predictive value; LR+, likelihood ratio for positive test; LR-, likelihood ratio for negative value.*

Of the 45 patients that yielded Rpfs-TLA positive outcomes with their pleural fluid samples, including 35 definite cases and 10 probable cases, the sensitivity of Rpfs-TLA was significantly different between the definite pleural TB 71.43% (35 of 49) and probable pleural TB 18.52% (10 of 54) groups (*p*-values < 0.001) (Table 2). When Rpfs-TLA outcomes were integrated into the composite reference standard, 10 of the 54 probable pleural TB cases were reclassified as definite cases, and the percentage of patients with definite pleural TB showed an obvious increase from 47.57% (49 of 103) to 57.28% (59 of 103). Of the 35 Rpfs-TLA positive cases in definite tuberculous pleurisy, there were 30 TLA positive cases, 27 Löwenstein–Jensen culture positive cases, and 9 smear positive cases, and no positive results of TLA, Löwenstein–Jensen culture, and smear in Rpfs-TLA negative cases (Figure 2).

### Mean Time of Positive Reporting

All of 35, 30, and 27 culture positive samples with Rpfs-TLA, TLA, and Löwenstein–Jensen were subjected to Ziehl–Neelsen staining and presented acid-fast bacilli positive. The monoclonal PCR products of each positive sample had IS6110 homologous sequences with H37Rv, confirmed to be *Mycobacterium tuberculosis*. The mean time of culture positive reporting were (11.87 ± 2.29 days) for Rpfs-TLA, (21.60 ± 3.33 days) for TLA, and (36.41 ± 4.02 days) for Löwenstein–Jensen, respectively. Both times of TLA and Löwenstein–Jensen were significantly longer than that of Rpfs-TLA (*F* = 539.49, *p*-value < 0.001). The mean culture positive reporting time of TLAs was longer than Löwenstein–Jensen culture, with significant statistical difference (*p*-value < 0.001).

### Mean Colony-Forming Units of Culture Positivity

The mean numbers of colony-forming units (CFUs) were (72.87 ± 48.71) by Rpfs-TLA and (28.17 ± 17.72) by TLA culture, and there was significant difference between TLA alone and the addition of Rpfs culture (*F* = 16.72, *p*-values < 0.001).

## Discussion

Tuberculous pleurisy is an exudative inflammation caused by *M.tb* directly affecting the pleura from the primary lesions near the pleura, or spreading to the pleura through blood and lymph circulation. A large amount of pleural effusion reduces the concentration of *M.tb*, and *M.tb* is dormant due to long-term lack of nutrition and oxygen, leading to extremely low positive rate of *M.tb* culture of pleural effusion *in vitro* (Liam et al., 2000; Mukamolova et al., 2010; O’Connor et al., 2015; Shaw et al., 2018; Dusthackeer et al., 2019; World Health Organization [WHO]., 2021). Rpfs are proteins with small molecular weight, originally identified in *Micrococcus luteus*, promoting the resuscitation of dormant bacilli to yield normal, viable colony-forming bacteria. The prototypes of Rpf were also present in other Gram-positive organism with high G + C content, like *M. tuberculosis* that contains five rpf-like genes (rpf A–E), which stimulated bacterial growth at picomole concentrations (Casalini et al., 2018). All Rpfs share a conserved domain of about 70 amino acids and possess a c-type lysozyme and lytic transglycosylases. The structures of the conserved domain suggest that Rpfs could be considered to restore the growth of dormant bacteria by cutting the peptidoglycan, which only exists on the cell wall of bacteria and has a physical inhibition effect on cell growth and division (Mukamolova et al., 1998, 2010; Biketov et al., 2000; Kana et al., 2008; Gupta and Srivastava, 2012; Rosser et al., 2017). Our preliminary experimental results have shown that Rpf B and Rpf E are highly expressed in *Escherichia coli* BL21 and are easy to be purified, and the resuscitation effect of the two Rpfs as supplement is equivalent to that of the five Rpfs A–E simultaneously. Therefore, we selected effective combinations of Rpf B and Rpf E from five Rpfs of *M.tb* as the supplement, expecting to improve culture performance.

In this study, the diagnostic performance of Rpfs-TLA (43.69%) demonstrated higher sensitivity than both TLA (29.13%, *p* < 0.005) and Löwenstein–Jensen culture (26.21%, *p* < 0.05), and all of the three culture methods were significantly higher than smear (*p* < 0.001). In addition, the mean numbers of CFUs by Rpfs-TLA was significantly higher than TLA. Mukamolova et al. (2010) reported that Rpfs could increase the yield of sputum culture. Some other studies showed that *M.tb* in sputum reveals a subpopulation of bacteria that do not grow in standard conditions but require an exogenous source of Rpfs for growth (Huang et al., 2014; Chengalroyen et al., 2016; Dusthackeer et al., 2019). This was also demonstrated in one extrapulmonary tuberculosis case (O’Connor et al., 2015). Although highly preliminary, these findings suggest that Rpfs could restore certain growth vitality of dormant and/or retained *M.tb* in pleural effusion, which could be the main reason for the improved sensitivity of Rpfs-TLA for paucibacillary TB diagnosis. In addition, Rpfs-TLA detected 10 additional positive outcomes from 54 probable pleural TB, which led to the percentage increase of patients with definite pleural TB from 47.57% (49 of 103) to 57.28% (59 of 103). The successful culture of *M.tb* from patient samples is essential for clinical practice, which could speed up the initiation of appropriate treatment and ascertaining drug susceptibility and strain typing (Gopi et al., 2007; Huang et al., 2014; O’Connor et al., 2015; Rosser et al., 2017).

Some reports demonstrated that TLA is rapid, easy to perform, and can be implemented as a reliable, economical alternative to conventional LJ medium and even to the automated MGIT 960 system for the diagnosis of pulmonary TB in low- to medium-volume laboratories (Idigoras et al., 1995; Martin et al., 2009a; Satti et al., 2010; Leung et al., 2012). Our study, for the first time, reports on the diagnostic accuracy of pleural effusion TLA for pleural TB. The sensitivity of TLA (29.13%) was similar to that of the Löwenstein–Jensen culture (26.21%). The mean time to results was 21.60 days for TLA, 11.87 days for Rpfs-TLA, and 36.41 days for Löwenstein–Jensen culture, indicating that Rpfs could resuscitate dormant bacteria in pleural effusion and speed up tuberculosis culture (Wu et al., 2008; Huang et al., 2014; Rosser et al., 2017; Dusthackeer et al., 2019). The mean report time of TLA was very close to that of MGIT 960 from pulmonary specimens, within the range of from 9.6 to 12.5 days (Martin et al., 2009b; Satti et al., 2010), but we have not compared with MGIT 960 in pleural TB, simultaneously. Besides, TLA showed comparable results to MGIT 960 for the rapid detection of *M.tb* drug susceptibility (DST) of second line anti-TB agents (Minion et al., 2010; Ardizzoni et al., 2015; Hussain et al., 2019). However, whether TLA supplemented with Rpfs could be suitable and promote clinical drug resistance detection still needs further study.

In this study, the liquefication treatment of clinical specimens should be carried out after centrifugation within 20 min, so as to avoid sodium hydroxide damage to the *M.tb* and affect the positive rate of culture. As the activity and purity of Rpf proteins is the key to the recovery and growth promotion effects of dormant bacteria, each batch of proteins should be identified for activity, concentration, and purity. Rpf B and Rpf E were added to the pretreated samples and fully mixed, then evenly coated on 7H11 medium for culture and colony count. Rpfs could cut the peptidoglycan, having a physical inhibition effect on cell growth and division, to restore the growth of dormant bacteria. In addition, the report time of *M.tb* can be shortened by observing the microcolonies of *M.tb* on TLA under inverted microscope and identifying them with colony characteristics and molecular biology. Whether the use of liquid medium for resuscitation culture can be better than BACTEC MGIT 960 liquid culture method and further shorten the reporting time is also worth further study.

In conclusion, Rpfs-TLA is a reliable, rapid, cheap, and simple method for the diagnosis of pleural tuberculous. As it does not need special and expensive instruments, it is promising for use in poor resource settings with high *M.tb* incidence. The study represents the first attempt to apply Rpfs promoting the growth of *M.tb* in pleural effusion, and more studies on a large scale are urgently needed to know its exact usefulness and accuracy.

## Data Availability Statement

The original contributions presented in the study are included in the article/supplementary material, further inquiries can be directed to the corresponding author/s.

## Ethics Statement

The studies involving human participants were reviewed and approved by the Beijing Chest Hospital Ethics Committee. The patients/participants provided their written informed consent to participate in this study.

## Author Contributions

ZZ and ZhL were the principal investigators who conceived the study and obtained financial support. FD, AX, ZiL, LP, ZhL, and ZZ designed the study. FD, AX, HJ, BD, QS, and RW were responsible for the recruitment of participants, collection of clinical information, and sample preparation. FD, AX, and BD conducted the data management and performed the statistical analyses. FD and AX interpreted the results and drafted the manuscript. All authors approved the final version of the manuscript.

## Conflict of Interest

The authors declare that the research was conducted in the absence of any commercial or financial relationships that could be construed as a potential conflict of interest.

## Publisher’s Note

All claims expressed in this article are solely those of the authors and do not necessarily represent those of their affiliated organizations, or those of the publisher, the editors and the reviewers. Any product that may be evaluated in this article, or claim that may be made by its manufacturer, is not guaranteed or endorsed by the publisher.
